# Urothelial genotoxicity of environmental chemicals detected in the urine of healthy dogs and their owners

**DOI:** 10.1017/cts.2024.546

**Published:** 2024-10-24

**Authors:** Hannah M. Peterson, Jenna C. Holler, Abby Boswell, Lauren A. Trepanier

**Affiliations:** Department of Medical Sciences, School of Veterinary Medicine, University of Wisconsin-Madison, Madison, USA

**Keywords:** Bladder cancer, acrolein, arsenic, environmental chemicals, urothelial carcinoma risk

## Abstract

**Introduction:**

Major risk factors for urothelial cell carcinoma (UCC) in people are smoking and occupational exposures. However, up to 30% of human UCC risk is still unexplained. Pet dogs develop UCC that models the clinical behavior of muscle-invasive human UCC. Dogs may therefore provide a useful model for non-tobacco, nonoccupational UCC risk. We previously found that nonsmoking human subjects and their pet dogs share exposures to the urothelial carcinogens acrolein and arsenic. We hypothesized that these urinary exposures would reach genotoxic concentrations in some individuals.

**Methods:**

We exposed immortal and primary human and canine urothelial cells *in vitro* to acrolein and inorganic arsenic and used the γ-H2AX and comet assays to measure DNA damage.

**Results:**

For acrolein, we found a genotoxic threshold of 1.1–4.4 μM in human cells and a threshold of 20.0–55.6 μM in canine cells. These findings are consistent with potentially genotoxic urinary acrolein exposures in 51% of healthy human subjects and 17% of pet dogs previously surveyed. For inorganic arsenic, we found a genotoxic threshold of ≥10 μM in canine and human cell lines. No healthy human or canine subject reached these urinary inorganic arsenic exposures when assayed at a single time point.

**Conclusions:**

Non-tobacco, nonoccupational acrolein exposures could increase the risk of early urothelial DNA damage in both people and pet dogs. Ongoing studies will assess these chemical exposures in the setting of UCC in both human and canine patients.

## Introduction

Urothelial cell carcinoma (UCC) is an environmental cancer in human patients. Up to 30% of cases are high-grade muscle invasive UCC, which has a 5-year mortality rate of about 50% [1]. Smoking and occupational exposures are known major risk factors, with only minor contributions from hereditary or medical factors [2]. Almost one-third of environmental risk for UCC is not well understood in human patients, which makes preventative measures difficult to implement. Part of this remaining UCC risk could be due to chronic chemical exposures in the household environment.

Pet dogs develop UCC that models the clinical presentation, molecular features, and poor prognosis of human muscle invasive UCC [3,4]. This makes the pet dog with UCC a potentially useful model for non-tobacco, nonoccupational UCC risk. Pet dogs develop UCC at an earlier absolute age of onset (median 11 years) [5]. compared to humans (median 65–70 years) [6]. This compressed life span, along with potentially intensified exposures compared with humans, could make it easier to identify relevant chemical exposure risk factors [7]. While some breeds of dogs have a higher risk for UCC [5], environmental factors can influence UCC development even among these high-risk breeds [8]. Environmental tobacco smoke has not been shown to be a risk factor for canine UCC [9,10], and occupational exposures are uncommon in pet dogs. Therefore, we hypothesize that household chemical exposures could be important in canine UCC risk and might be a relevant model for non-tobacco, nonoccupational bladder cancer risk in people.

**Figure 1.**
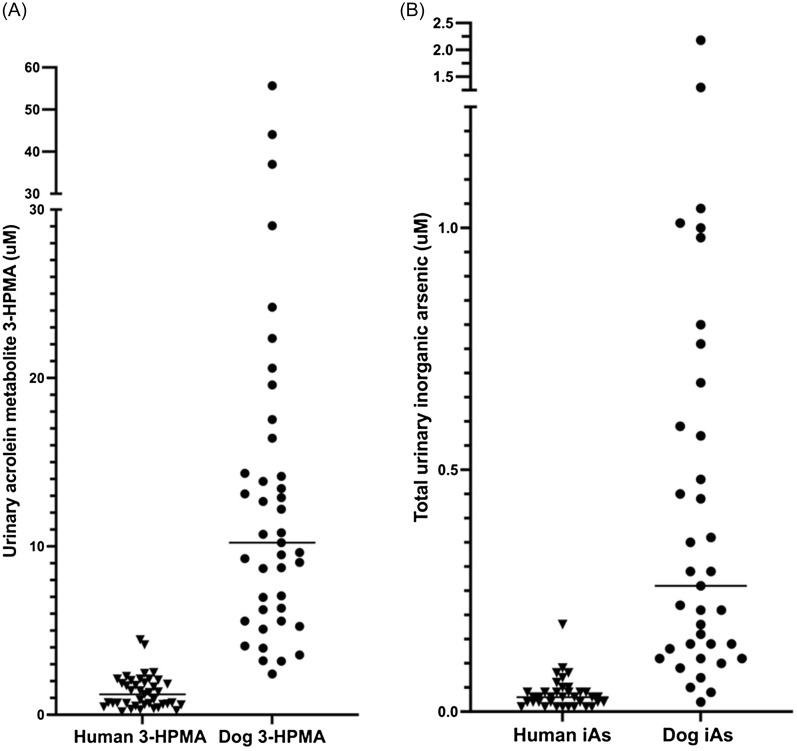
Previously measured acrolein (as its stable metabolite, 3-HPMA; **Panel A**) and urinary inorganic arsenic (iAs; the sum of As [III], As[V], dimethylarsinic acid, and monomethylarsonic acid; **Panel B**) in the urine of healthy pet dogs and their owners [11].

As a first step in assessing relevant household chemical exposures in dogs and people, we measured concentrations of several known or suspected urothelial carcinogens in the urine of healthy pet dogs and their owners [11]. We found that urinary metabolites of acrolein and inorganic arsenic were readily detectable in both species (Figure 1) and were correlated across dogs and their owners in the same household, which suggested shared exposure sources [11]. We further hypothesize that urinary chemical exposures to acrolein and inorganic arsenic might reach genotoxic concentrations in some healthy dogs and their owners.

The aim of this study was to determine whether acrolein and inorganic arsenic exposures, previously observed as stable metabolites in the urine of healthy pet dogs and their owners, reached genotoxic concentrations in some individuals, when tested *in vitro* in canine and human urothelial cell lines using the γ-H2AX and comet assays as measures of DNA damage.

## Materials and Methods

### Cell culture of immortalized urothelial cell lines

Canine K9TCC-AxC and K9TCC-SH cells, derived from two female dogs with intermediate to high-grade muscle-invasive UCC [12], were a gift from Dr Deborah Knapp at Purdue University. Fetal bovine serum (FBS) was purchased from Sigma-Aldrich. Dulbecco’s modified Eagle medium nutrient mix (DMEM) and dimethyl sulfoxide (DMSO) were purchased from Fisher Scientific. The cells were maintained in DMEM with L-glutamine and phenol red supplemented with 10% FBS. Cells were at passage 35 (K9TCC-AxC) and 39 (K9TCC-SH) when received and were expanded to passages 42 and 44, respectively, and banked in liquid nitrogen with 10% DMSO prior to experimental use.

Human adherent urothelial carcinoma cell lines, HT-1376 (CRL-1472^TM^) and T24 (HTB-4^TM^), were purchased from ATCC (Manassas, VA) and maintained in minimum essential medium (MEM) with phenol red supplemented with 10% FBS and L-glutamine at a final concentration of 2 mM or McCoy’s 5A Medium modified with 10% FBS, respectively. Cells were received at passages 43 (HT-1376) and 38 (T24) were grown to passages 50 and 45, respectively, and were banked in 10% DMSO in liquid nitrogen prior to experimental use. L-glutamine and MEM were purchased from Sigma-Aldrich. McCoy’s 5A medium was purchased from Fisher Scientific.

Prior to chemical exposures, urothelial cells were thawed and passaged twice to ∼70% confluence. Experiments were performed at passage 44 for K9TCC-AxC, passage 46 for K9TCC SH, passage 52 for HT-1376, and passage 47 for T24 cells. Cells destined for the γ-H2AX assay were counted and transferred to chamber slides (Lab-Tek II 4-Well Chamber Glass Slides, Thermo Fisher, Rochester, NY) at 2.5 × 10^4^ cells/well and left overnight for 18 h to adhere, in an incubation chamber at 37 °C with 5% CO_2_. Cells used for the CometChip assay were immediately utilized after reaching ∼70% confluence.

### Cell culture of primary urothelial cell lines

Canine primary urothelial cells were obtained from Creative Bioarray (Cat No: CSC-C9111J, Donor ID 363633) and maintained in SuperCult® bladder epithelial cell medium (Cat No: CM-C9111J). Cells were received at passage 2, seeded in gelatin-coated flasks at 5,000 cells per cm^2^, and grown to passage 4 before immediate use in experiments.

Human primary urothelial cells were obtained from ATCC (Cat No: PCS-420-010, Donor ID 80203232). They were maintained in complete bladder epithelial cell basal medium supplemented with growth kit components purchased from ATCC (Cat No: PCS-420-032 and PCS-420-042). Cells were received at passage 2, seeded at 5,000 cells per cm^2^, and expanded to passage 3 before immediate use in experiments.

### Chemical exposures

Previous urinary chemical concentrations in 42 healthy dogs and their 42 owners, reported in ng/mg creatinine [11], were converted to direct μM concentrations based on each chemical’s molecular weight. Urinary exposures to acrolein were extrapolated from its measured stable urine metabolite, 3-hydroxypropylmercapturic acid (3-HPMA), on an equivalent molar basis (Figure 1A) since 3-HPMA was not available commercially. Urinary exposures to inorganic arsenic were determined as the molar sum of all measured inorganic arsenic species: iAs(V), iAs (III), dimethylarsinic acid, and monomethylarsinic acid (Figure 1B) [11].

Human and canine immortal and primary urothelial cells were incubated with either acrolein or with inorganic arsenic as sodium arsenite. Acrolein was procured from Restek (Bellafonta, Pennsylvania), while sodium arsenite was purchased from Sigma-Aldrich (St. Louis, Missouri). For each chemical and cell line combination, time course experiments were performed over 24 h at 37 °C (5% CO_2_) to determine time to peak genotoxicity at the highest observed *in vivo* urinary concentrations for each species. Concentration-dependent genotoxicity experiments, encompassing the range of observed urinary concentrations, were then performed at these peak times for each chemical/cell line combination. Peak genotoxicity was observed at 6 h for all cell lines with either acrolein or iAs (III) (data not shown). Each concentration-dependent genotoxicity experiment was performed in triplicate on at least two separate occasions.

### Immunocytochemistry for γ-H2AX

For immortal cell lines, DNA damage was assessed using both the γ-H2AX assay, which detects a histone protein, H2AX, that is, phosphorylated in response to single- and double-stranded DNA damage [13]. and the comet assay, which detects single- and double-stranded DNA damage more directly.

Cells destined for the γ-H2AX assay were washed after chemical incubations with prewarmed 1X phosphate- buffered solution (PBS), fixed on slides with 4% formaldehyde, and washed in 1X PBS. Slides were kept at 4^o^C for no more than 1 week prior to the γ-H2AX assay.

Immunocytochemistry for γ-H2AX was performed with an anti-phospho-histone H2A.X [Ser139] mouse monoclonal antibody (Abcam, Cambridge, MA) and an anti-mouse IgG Fab2 Alexa Fluor® 488 antibody (Cell Signaling Technology, Danvers, MA), as previously described [14]. Slides were counterstained with DAPI (Thermo Scientific, Rockford, IL) to detect cell nuclei. Imaging for γ-H2AX foci was performed with a Leica TCS SP8 laser scanning confocal microscope (Leica Microsystems, Wetzlar, Germany), equipped with a 63x oil-immersion objective. Two channels were acquired sequentially with the following parameters: 488 nm for the secondary antibody and 405 nm for DAPI [14]. The γ-H2AX foci were quantified using open access software (FoCo, MATLAB), which is robust and reliable for automated counting of nuclear foci in single-cell images [15]. At least 100 cells were counted per replicate, and data were expressed as mean γ-H2AX foci/nucleus.

### Alkaline CometChip assay

We also assessed DNA damage using the traditional alkaline comet assay, using a high-throughput adaptation, the CometChip [16]. The CometChip uses a 96 well format that contains hundreds of microwells per well to improve cell distribution, reduce cell clumping, and minimize inter- and intra-assay variability [16]. We fabricated CometChips using a 30-micron stamp gifted to our lab from Dr Bevin Engelward at the Massachusetts Institute of Technology, using a protocol developed in her laboratory [16].

In preparation for the CometChip assay, immortal or primary urothelial cells were counted and passed through a 40 mm filter to remove any clumps. Then 100 mL of cell solution (∼100,000 cells) was added to each well. Cells were incubated at 37°C with 5% CO_2_ for 30 min to allow them to settle into the microwells. Wells were washed with 1X PBS, overlaid with 1% low-melting point agarose in 1X TBE buffer, and allowed to solidify. Concentrations of sodium arsenite or acrolein were added to each well and incubated for 6 h for each cell line. After dosing, chips were immersed in an alkaline lysis solution (2.5 M NaCl, 100 mM Na_2_EDTA, 10 mM Trizma® Base, and 1% Triton X-100, adjusted to pH 10) at 4°C overnight. Chips were then immersed in an alkaline unwinding buffer (0.3 M NaOH and 1 mM Na_2_EDTA) for 40 min at 4°C. The DNA was electrophoresed in the same buffer for 30 min at 1 V/cm and ∼ 300 mA. Chips were submerged twice in neutralization buffer (0.4 M Trizma® base at pH 7.5) for 15 min each, washed with deionized water and then 70% ethanol, and dried overnight before imaging.

Cellular DNA in the CometChip wells was stained for 30 min at room temperature with 2X SYBR™ Gold in 1X TBE buffer. Imaging was performed with the Leica TCS SP8 laser scanning confocal microscope with a 4X dry objective with a 495 nm excitation filter. Percent DNA in the comet tails were quantified using CometAssay Analysis Software (Bio-Techne, Minneapolis, MN). At least 50 cells were counted per replicate. Each experiment was performed in triplicate on two separate occasions. Data were expressed as mean percent DNA in the comet tail [17].

## Statistical analyses

DNA damage as mean γ-H2AX foci/nucleus or percent tail DNA was compared between vehicle and chemical concentrations using one-way ANOVA, followed by Dunnett’s multiple comparison tests. Statistical analyses were performed using commercial software (Prism 9, GraphPad Software LLC). *P* < 0.05 was considered statistically significant.

## Results

### Acrolein

**Figure 2.**
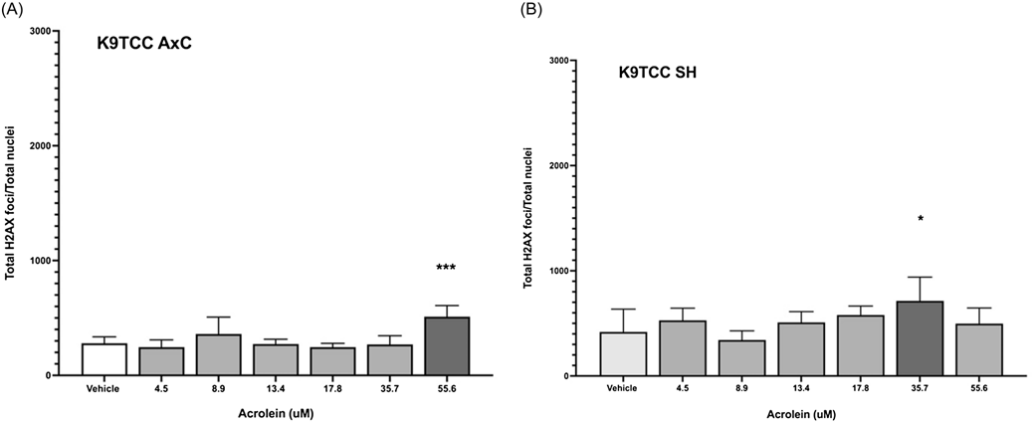
Genotoxicity of acrolein using the γ-H2AX assay in canine urothelial cell lines K9TCC-AxC (**Panel A**; *** *P* = 0.0001) and K9TCC-SH (**Panel B**; * *P* = 0.014).

The highest extrapolated acrolein exposure previously observed in healthy canine urine was 55.6 μM (12,300 ng/mL 3-HPMA; Figure 1A). Using the γ-H2AX assay, acrolein led to significant DNA damage over vehicle at 55.6 μM in K9TCC-AxC cells (Figure 2A) and at 35.7 μM in K9TCC SH cells (7,892 ng/mL 3-HPMA; Figure 2B).

**Figure 3.**
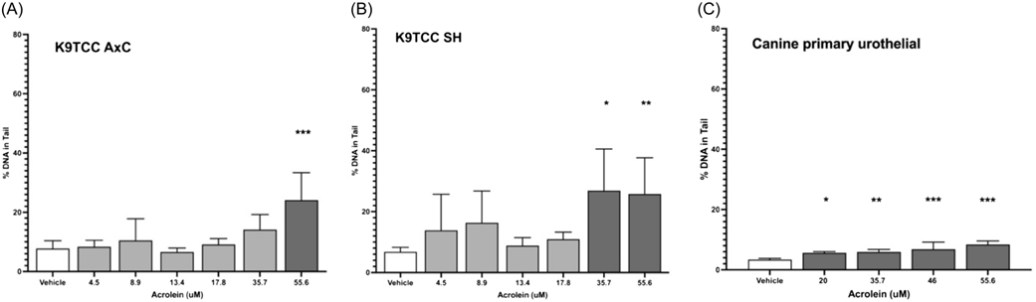
Genotoxicity of acrolein using the CometChip assay in canine urothelial cell lines K9TCC-AxC (**Panel A**; *** *P* = 0.0001) and K9TCC-SH (**Panel B**; * *P* = 0.013; ** *P* = 0.009), and in primary canine urothelial cells (**Panel C**; * *P* = 0.02; ** *P* = 0.006; *** *P* ≤ 0.004).

Using the comet assay, the same thresholds for DNA damage were observed in the canine immortal cell lines (55.6 μM for K9TCC-AxC cells (Figure 3A) and 35.7 μM for K9TCC SH cells (Figure 3B). The genotoxic threshold for acrolein in primary canine urothelial cells was 20.0 μM (Figure 3C). Using 20 μM as a threshold for acrolein genotoxicity, 7 of 41 healthy dogs (17%) previously screened [11] had potentially genotoxic urinary exposures to acrolein at a single time point (Figure 1A).

**Figure 4.**
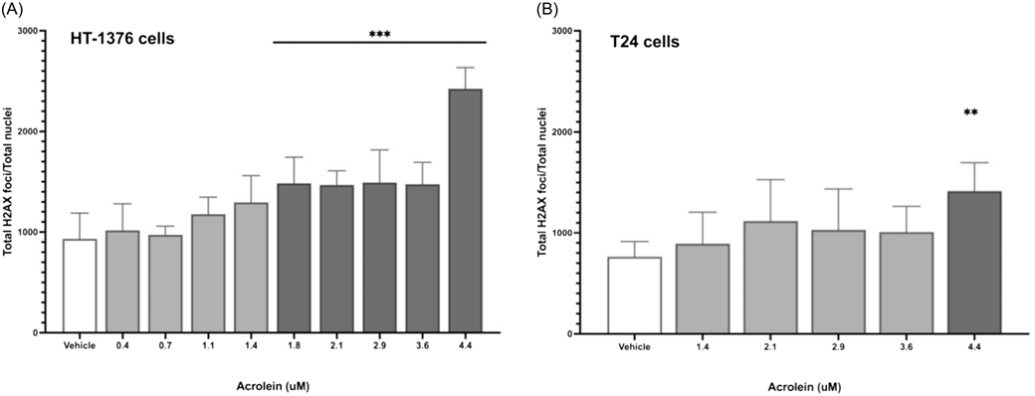
Genotoxicity of acrolein using the γ-H2AX assay in human urothelial cell lines HT-1376 (**Panel A**; *** *P* ≤ 0.0009) and T24 (**Panel B**; ** *P* = 0.008).

In healthy human urine [11], the highest extrapolated acrolein exposure was 4.5 μM (984 ng/mL 3-HPMA; Figure 1A). Using the γ-H2AX assay, acrolein showed significant concentration-dependent DNA damage over vehicle at concentrations ≥1.8 μM (394 ng/mL 3-HPMA) in HT-1376 cells (Figure 4A), and at 4.4 μM (973 3-HPMA ng/mL) in T24 cells (*P* = 0.008; Figure 4B).

**Figure 5.**
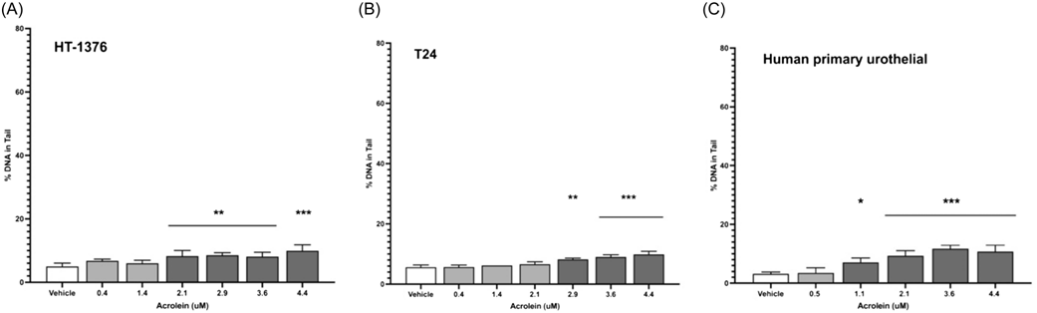
Genotoxicity of acrolein using the CometChip assay in human urothelial cell lines HT-1376 (**Panel A**; ** *P* ≤ 0.005; *** *P* < 0.0001) and T24 (**Panel B**; ** *P* = 0.003; *** *P* < 0.0001), and in human primary urothelial cells (**Panel C**; * *P* = 0.007; *** *P* < 0.0001).

Using the comet assay, DNA damage thresholds for acrolein were ≥2.1 μM for HT-1376 cells and ≥2.9 μM for T24 cells (Figure 5A and 5B). The threshold for acrolein genotoxicity in primary human urothelial cells was ≥1.1 μM (Figure 5C). Using 1.1 μM as a possible threshold for genotoxicity, 21 of 41 human subjects previously screened (51%) had potentially genotoxic urinary exposures to acrolein at a single time point (Figure 1A).

### Total inorganic arsenic

In initial experiments performed with urothelial cells exposed to iAs and assayed for DNA damage using the γ-H2AX assay, arsenic-induced DNA damage was not detectable (data not shown). On review of the literature, we found that iAs can indirectly inhibit H2AX phosphorylation by blocking upstream activation of the DNA repair protein ataxia telangiectasia mutated (ATM) [18]. Therefore, we are only reporting DNA damage from iAs using the alkaline CometChip assay, which directly assesses DNA strand damage without need for activation of this pathway.

**Figure 6.**
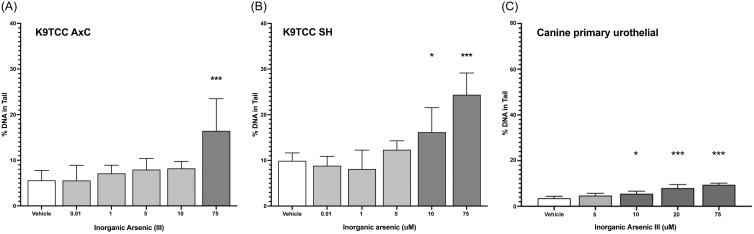
Genotoxicity of inorganic arsenic (as sodium arsenite) using the CometChip assay in canine urothelial cell lines K9TCC-AxC (**Panel A**; *** *P* < 0.0001) and K9TCC-SH (**Panel B**; * *P* = 0.026; *** *P* < 0.0001), and in canine primary urothelial cells (**Panel C;** * *P* = 0.04; *** *P* < 0.0001).

Total measured inorganic arsenic species in healthy canine urine ranged from 0.02 to 2.18 μM (Figure 1B). Using the comet assay, inorganic arsenic led to significant DNA damage compared to vehicle at ≥10 μM in K9TCC-SH cells and only at 75 μM in K9TCC-AxC cells, (Figure 6A–B). In primary canine urothelial cells, the genotoxic threshold for inorganic arsenic was also 10 μM (Figure 6C**).** No healthy dogs reached this urinary inorganic arsenic concentration when screened at a single time point.

**Figure 7.**
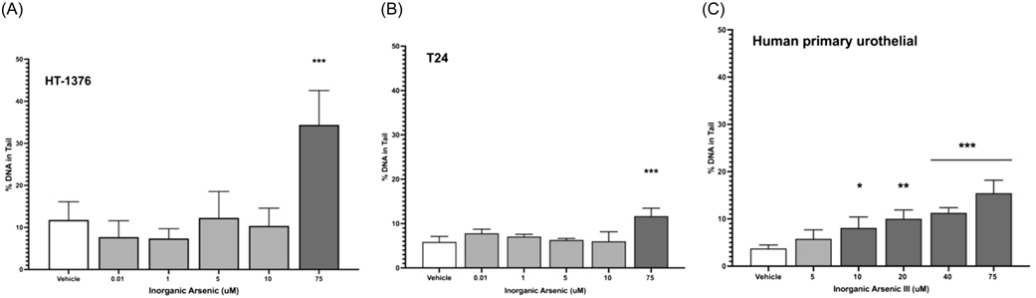
Genotoxicity of inorganic arsenic (as sodium arsenite) using the CometChip assay in human urothelial cell lines HT-1376 (**Panel A**) and T24 (**Panel B**) *** *P* < 0.0001, and in human primary urothelial cells (**Panel C;** * P = 0.005; ** P = 0.0006; *** P < 0.0001).

In healthy human subjects, total measured urinary inorganic arsenic ranged from 0.01 to 0.18 μM (Figure 1B). Inorganic arsenic led to significant DNA damage compared to vehicle only at 75 μM in both immortal human cell lines (*P* < 0.0001; Figure 7A and B) and at 10 μM in human primary urothelial cells (Figure 7C). No healthy human subjects reached this urinary inorganic arsenic concentration when screened at a single time point.

## Discussion

In our previous study, we found that healthy pet dogs had significantly higher urinary concentrations of the acrolein metabolite 3-HPMA and total inorganic arsenic compared to their human owners sharing the same household, with wide individual variability within species [11]. The goal of the current study was to assess the genotoxic potential of these urinary exposures in both dogs and people.

Acrolein was genotoxic to immortal and primary human urothelial cell lines with thresholds of 1.8 and 1.1 μM, respectively. These data are consistent with a previously reported threshold of 2.5 μM acrolein for mutagenic transformation of human urothelial cells [19]. In our immortal and primary canine urothelial cell lines, acrolein had a genotoxic threshold of ≥20 μM, which was nearly 20-fold higher than in human urothelial cells. It is unclear whether canine urothelial cells are truly less sensitive to DNA damage from acrolein *in vivo*, or whether this is sampling bias from the cell lines chosen. While median acrolein exposures were more than eightfold higher in pet dogs (Figure 1), predicted *in vivo* genotoxic exposures were reached in a relatively lower proportion of dogs (17%) compared to people (51%).

We used urinary exposures to the stable metabolite 3-HPMA as a surrogate for urinary acrolein exposures. However, it is unclear whether 3-HPMA concentrations reflect direct urothelial exposures to acrolein, and because of its volatility, acrolein itself cannot be directly measured. Acrolein does reach urothelial cells however, as genotoxic and mutagenic DNA adducts have been detected in healthy human bladder mucosa [20]. Follow-up studies could include measurement of acrolein–protein conjugates in the peripheral blood of enrolled subjects or acrolein-DNA adducts in bladder biopsy samples.

For inorganic arsenic, we found that the γ-H2AX assay was insensitive to DNA damage from iAs. We attribute this to the observation that iAs inhibits ATM, a vital component of the pathway that leads to phosphorylation of H2AX [18], which would lead to false-negative results from iAs in the γ-H2AX assay. We, therefore, utilized the alkaline CometChip assay to directly measure both single- and double-stranded DNA breaks and added additional parallel experiments using the CometChip assay for acrolein [16]. We found that inorganic arsenic reached a genotoxic threshold of 10 μM in K9TCC-SH cells and in both primary canine and human urothelial cell lines. Similarly, a genotoxic threshold of 10 μM was reported for sodium arsenite in human HUC-RAS cells [21], whereas concentrations of 1–10 μM led to increases in urothelial p53 mutations [22]. No healthy canine or human subjects reached the 10 μM urinary threshold in our previous *in vivo* study [11].

In another study, sodium arsenite led to modest but significant increases in comet tails in human SV-HUC urothelial cells at 1 μM, albeit with a 48-h incubation period [22]. Using a 1 μM threshold, no human subjects, but 5 of 37 healthy dogs (∼14%) would have had potentially genotoxic urothelial exposures to iAs.

Limitations of these experiments include the use of only one primary and two immortal cell lines per species and the uncertainties of extrapolation from cell lines to urothelial cells *in vivo*. Immortal cell lines do not replicate *in vivo* heterogeneity and can vary greatly in their gene and protein expression patterns [23,24], which could include differences in DNA repair mechanisms. Primary cells better represent normal urothelium but may not generate a normal urothelial barrier. Follow-up studies could further incorporate human and canine urinary organoids, which may better recapitulate the cellular heterogeneity and biologic behavior of urothelial cells of origin [25,26]. In addition, indirect mechanisms of chemically induced DNA damage, such as oxidative stress, could be evaluated by adding formamidopyrimidine DNA glycosylase prior to electrophoresis in the CometChip assay [27]. This additional step unveils sites of oxidative damage to purines, which may occur independently of DNA strand breaks [27]. Finally, our experiments reflect only acute chemical exposures, and longer incubation of urothelial cells with acrolein or arsenic would better reflect the effects of chronic *in vivo* urothelial exposures.

Overall, the results of this study suggest that healthy individuals and their pet dogs, in the absence of known occupational exposures, may have potentially genotoxic concentrations of acrolein in their urine, which could increase the risk of urothelial DNA damage. We are currently assessing urinary acrolein and inorganic arsenic levels in casecontrol studies in both human patients and pet dogs with UCC. The overall goal of these studies is to identify non-tobacco, non-occupational sources of exposure to genotoxic urothelial carcinogens in the household, so that evidence-based prevention measures can be considered.

## References

[1] Zhang J , Zhou X , Ding H , et al. The prognostic value of routine preoperative blood parameters in muscle-invasive bladder cancer. BMC Urol. 2020; 20: 31. doi: 10.1186/s12894-020-00602-9. 32192483 PMC7082918

[2] Czerniak B , Dinney C , McConkey D. Origins of bladder cancer. Annu Rev Pathol. 2016; 11: 149–174.doi: 10.1146/pathmechdis.2016.11. 26907529

[3] Knapp DW , Ramos-Vara JA , Moore GE , Dhawan D , Bonney PL , Young KE. Urinary bladder cancer in dogs, a naturally occurring model for cancer biology and drug development. ILAR J. 2014; 55: 100–118. doi: 10.1093/ilar/ilu018. 24936033

[4] Knapp DW , Dhawan D , Ramos-Vara JA , et al. Naturally-occurring invasive urothelial carcinoma in dogs, a unique model to drive advances in managing muscle invasive bladder cancer in humans. Front Oncol. 2020; 9: 1493–1493. doi: 10.3389/fonc.2019.01493. 32039002 PMC6985458

[5] Knapp DW , Glickman NW , DeNicola DB , Bonney PL , Lin TL , Glickman LT. Naturally-occurring canine transitional cell carcinoma of the urinary bladder a relevant model of human invasive bladder cancer. Urol Oncol. 2000; 5: 47–59. doi: 10.1016/S1078-1439(99)00006-X. 21227289

[6] Kirkali Z , Chan T , Manoharan M , et al. Bladder cancer: epidemiology, staging and grading, and diagnosis. Urology. 2005; 66: 4–34. doi: 10.1016/j.urology.2005.07.062. 16399414

[7] Knapp DW , Dhawan D , Ostrander E. “Lassie,” “toto,” and fellow pet dogs: poised to lead the way for advances in cancer prevention. ASCO. 35: 2015; e667–e672. 10.14694/EdBook_AM.2015.35.e667 25993240

[8] Glickman LT , Raghavan M , Knapp DW , Bonney PL , Dawson MH. Herbicide exposure and the risk of transitional cell carcinoma of the urinary bladder in scottish terriers. J Am Vet Med Assoc. 2004; 15: 1290–1297. doi: 10.2460/javma.2004.224.1290. 15112777

[9] Glickman LT , Schofer FS , McKee LJ , Reif JS , Goldschmidt MH. Epidemiologic study of insecticide exposures, obesity, and risk of bladder cancer in household dogs. J Toxicol Environ Health. 1989; 28: 407–414. doi: 10.1080/15287398909531360. 2593174

[10] Luethcke KR , Ekena J , Chun R , Trepanier LA. Glutathione s-transferase theta genotypes and environmental exposures in the risk of canine transitional cell carcinoma. J Vet Intern Med. 2019; 33: 1414–1422. doi: 10.1111/jvim.15504. 31008543 PMC6524089

[11] Craun K , Luethcke KR , Shafer M , et al. Environmental chemical exposures in the urine of dogs and people sharing the same households. J Clin Transl Sci. 2020; 5: e54. doi: 10.1017/cts.2020.548. 33948275 PMC8057441

[12] Dhawan D , Ramos-Vara JA , Stewart JC , Zheng R , Knapp DW. Canine invasive transitional cell carcinoma cell lines: in vitro tools to complement a relevant animal model of invasive urinary bladder cancer. Urol Oncol. 2009; 27: 284–292. doi: 10.1016/j.urolonc.2008.02.015. 18562222

[13] Kopp B , Khoury L , Audebert M. Validation of the γH2AX biomarker for genotoxicity assessment: a review. Arch Toxicol. 2019; 93: 2103–2114. doi: 10.1007/s00204-019-02511-9. 31289893

[14] Peterson HM , Manley CI , Trepanier LA , Pritchard JC. Genotoxicity from metronidazole detected in vitro, but not in vivo, in healthy dogs in a randomized clinical trial. Am J Vet Res. 2022; 84: 1–6. doi: 10.2460/ajvr.22.07.0112, 36346697

[15] Lapytsko A , Kollarovic G , Ivanova L , Studencka M , Schaber J. FoCo: a simple and robust quantification algorithm of nuclear foci. BMC Bioinform. 2015; 16: 392. doi: 10.1186/s12859-015-0816-5. PMC4654864 26589438

[16] Sykora P , Chiari Y , Heaton A , Moreno N , Glaberman S , Sobol RW. Application of the cometchip platform to assess DNA damage in field-collected blood samples from turtles. Environ Mol Mutagen. 2018; 59: 322–333. doi: 10.1002/em.22183. 29536573 PMC5902651

[17] Collins AR. The comet assay for DNA damage and repair: principles, applications, and limitations. Mol Biotechnol. 2004; 26: 249–261. 15004294 10.1385/MB:26:3:249

[18] Nail AN , McCaffrey LM , Banerjee M , Ferragut Cardoso AP , States JC. Chronic arsenic exposure suppresses ATM pathway activation in human keratinocytes. Toxicol Appl Pharmacol. 2022;Jul 1 446:116042–116042. doi: 10.1016/j.taap.2022. 35513056 PMC9318262

[19] Lee HW , Wang HT , Weng MW , et al. Cigarette side-stream smoke lung and bladder carcinogenesis: inducing mutagenic acrolein-DNA adducts, inhibiting DNA repair and enhancing anchorage-independent-growth cell transformation. Oncotarget. 2015; 6: 33226–33236. doi: 10.18632/oncotarget.5429. 26431382 PMC4741761

[20] Lee H-W , Wang H-T , Weng M-W et al. Acrolein- and 4-aminobiphenyl-DNA adducts in human bladder mucosa and tumor tissue and their mutagenicity in human urothelial cells. Oncotarget. 2014; 5: 3526–3540. doi: 10.18632/oncotarget.v5i11. 24939871 PMC4116500

[21] Liao Y-C , Chen Y-F , Lee T-C. Increased susceptibility of H-RasG12V-transformed human urothelial cells to the genotoxic effects of sodium arsenite. Arch Toxicol. 2015; 89: 1971–1979. 25199681 10.1007/s00204-014-1344-1

[22] Chai CY , Huang YC , Hung WC , Kang WY , Chen WT. Arsenic salt-induced DNA damage and expression of mutant p53 and COX-2 proteins in SV-40 immortalized human uroepithelial cells. Mutagenesis. 2007; 22: 403–408. doi: 10.1093/mutage/gem035. 17906315

[23] Pinto-Leite R , Carreira I , Melo J , et al. Genomic characterization of three urinary bladder cancer cell lines: understanding genomic types of urinary bladder cancer. Tumour Biol. 2014; 35: 4599–4617. doi: 10.1007/s13277-013-1604-3. 24459064

[24] Zuiverloon TCM , de Jong FC , Costello JC , Theodorescu D. Systematic review: characteristics and preclinical uses of bladder cancer cell lines. Bladder Cancer. 2018; 26: 169–183. doi: 10.3233/blc-180167. PMC5929350 29732388

[25] Elbadawy M , Usui T , Mori T , et al. Establishment of a novel experimental model for muscle-invasive bladder cancer using a dog bladder cancer organoid culture. Cancer Sci. 2019; 110: 2806–2821. doi: 10.1111/cas.v110.9. 31254429 PMC6726682

[26] Minoli M , Cantore T , Hanhart D , et al. Bladder cancer organoids as a functional system to model different disease stages and therapy response. Nat Commun. 2023; 14: 2214. doi: 10.1038/s41467-023-37696-2. 37072390 PMC10113240

[27] Marsà A , Cortés C , Hernández A , Marcos R. Hazard assessment of three haloacetic acids, as byproducts of water disinfection, in human urothelial cells. Toxicol Appl Pharmacol. May 15 2018; 347: 70–78. doi: 10.1016/j.taap.2018.04.004. 29634955

